# Can apparent diffusion coefficient (ADC) distinguish breast cancer from benign breast findings? A meta-analysis based on 13 847 lesions

**DOI:** 10.1186/s12885-019-6201-4

**Published:** 2019-10-15

**Authors:** Alexey Surov, Hans Jonas Meyer, Andreas Wienke

**Affiliations:** 1 Department of Diagnostic and Interventional Radiology, University of Leipzig, Liebigstr. 20, 04103 Leipzig, Germany; 2 Department of Diagnostic and Interventional Radiology, Ulm University Medical Center, Albert-Einstein-Allee 23, 89081 Ulm, Germany; 3 Institute of Medical Epidemiology, Biostatistics, and Informatics, Martin-Luther-University Halle-Wittenberg, Magdeburger Str. 8, 06097 Halle, Germany

**Keywords:** Breast cancer, ADC, MRI

## Abstract

**Background:**

The purpose of the present meta-analysis was to provide evident data about use of Apparent Diffusion Coefficient (ADC) values for distinguishing malignant and benign breast lesions.

**Methods:**

MEDLINE library and SCOPUS database were screened for associations between ADC and malignancy/benignancy of breast lesions up to December 2018. Overall, 123 items were identified. The following data were extracted from the literature: authors, year of publication, study design, number of patients/lesions, lesion type, mean value and standard deviation of ADC, measure method, b values, and Tesla strength.

The methodological quality of the 123 studies was checked according to the QUADAS-2 instrument. The meta-analysis was undertaken by using RevMan 5.3 software. DerSimonian and Laird random-effects models with inverse-variance weights were used without any further correction to account for the heterogeneity between the studies. Mean ADC values including 95% confidence intervals were calculated separately for benign and malign lesions.

**Results:**

The acquired 123 studies comprised 13,847 breast lesions. Malignant lesions were diagnosed in 10,622 cases (76.7%) and benign lesions in 3225 cases (23.3%). The mean ADC value of the malignant lesions was 1.03 × 10^− 3^ mm^2^/s and the mean value of the benign lesions was 1.5 × 10^− 3^ mm^2^/s. The calculated ADC values of benign lesions were over the value of 1.00 × 10^− 3^ mm^2^/s. This result was independent on Tesla strength, choice of b values, and measure methods (whole lesion measure vs estimation of ADC in a single area).

**Conclusion:**

An ADC threshold of 1.00 × 10^− 3^ mm^2^/s can be recommended for distinguishing breast cancers from benign lesions.

## Background

Magnetic resonance imaging (MRI) plays an essential diagnostic role in breast cancer (BC) [1, 2]. MRI has been established as the most sensitive diagnostic modality in breast imaging [1–3]. Furthermore, MRI can also predict response to treatment in BC [4]. However, it has a high sensitivity but low specificity [5]. Therefore, MRI can often not distinguish malignant and benign breast lesions. Numerous studies reported that diffusion-weighted imaging (DWI) has a great diagnostic potential and can better characterize breast lesions than conventional MRI [6–8]. DWI is a magnetic resonance imaging (MRI) technique based on measure of water diffusion in tissues [9]. Furthermore, restriction of water diffusion can be quantified by apparent diffusion coefficient (ADC) [9, 10]. It has been shown that malignant tumors have lower values in comparison to benign lesions [7]. In addition, according to the literature, ADC is associated with several histopathological features, such as cell count and expression of proliferation markers, in different tumors [11, 12].

However, use of ADC for discrimination BC and benign breast lesions is difficult because of several problems. Firstly, most reports regarding ADC in several breast cancers and benign breast lesions investigated relatively small patients/lesions samples. Secondly, the studies had different proportions of malignant and benign lesions. Thirdly and most importantly, the reported ADC threshold values and as well specificity, sensitivity, and accuracy values ranged significantly between studies. For example, in the study of Aribal et al., 129 patients with 138 lesions (benign *n* = 63; malignant *n* = 75) were enrolled [13]. The authors reported the optimal ADC cut-off as 1.118 × 10^− 3^ mm2/s with sensitivity and specificity 90.67, and 84.13% respectively [13]. In a study by Arponen et al., which investigated 112 patients (23 benign and 114 malignant lesions), the ADC threshold was 0.87 × 10^− 3^ mm^2^/s with 95.7% sensitivity, 89.5% specificity and overall accuracy of 89.8% [14]. Cakir et al. reported in their study with 52 women and 55 breast lesions (30 malignant, 25 benign) an optimal ADC threshold as ≤1.23 × 10^− 3^ mm^2^/s (sensitivity = 92.85%, specificity = 54.54%, positive predictive value = 72.22%, negative predictive value = 85.71%, and accuracy = 0.82) [15]. Finally, different MRI scanners, Tesla strengths and b values were used in the reported studies, which are known to have a strong influence in ADC measurements. These facts question the possibility to use the reported ADC thresholds in clinical practice.

To overcome these mentioned shortcomings, the purpose of the present meta-analysis was to provide evident data about use of ADC values for distinguishing malignant and benign breast lesions.

## Methods

### Data acquisition and proving

**Fig. 1:**
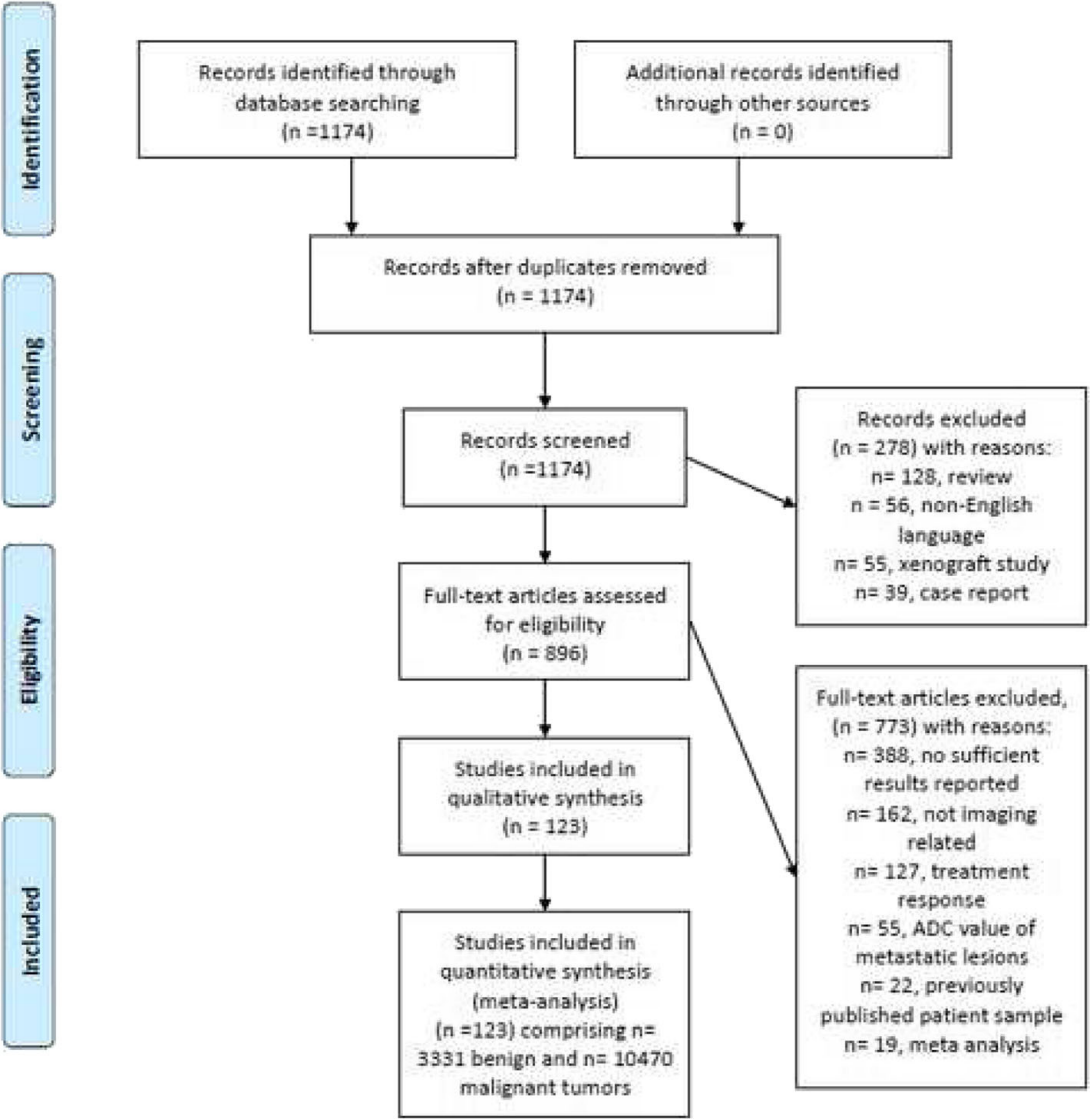
PRISMA flow chart of the data acquisition

Figure 1 shows the strategy of data acquisition. MEDLINE library and SCOPUS database were screened for associations between ADC and malignancy/benignancy of breast lesions up to December 2018. The following search terms/combinations were as follows:

“DWI or diffusion weighted imaging or diffusion-weighted imaging or ADC or apparent diffusion coefficient AND breast cancer OR breast carcinoma OR mammary cancer OR breast neoplasm OR breast tumor”. Secondary references were also manually checked and recruited. The Preferred Reporting Items for Systematic Reviews and Meta-Analyses statement (PRISMA) was used for the research [16].

Overall, the primary search identified 1174 records. The abstracts of the items were checked. Inclusion criteria for this work were as follows:

- Data regarding ADC derived from diffusion weighted imaging (DWI);
- Available mean and standard deviation values of ADC;
- Original studies investigated humans;
- English language.

Overall, 127 items met the inclusion criteria. Other 1017 records were excluded from the analysis. Exclusion criteria were as follows:

- studies unrelated to the research subjects;
- studies with incomplete data;
- non-English language;
- duplicate publications;
- experimental animals and in vitro studies;
- review, meta-analysis and case report articles;

The following data were extracted from the literature: authors, year of publication, study design, number of patients/lesions, lesion type, mean value and standard deviation of ADC, and Tesla strength.

### Meta-analysis

**Fig. 2:**
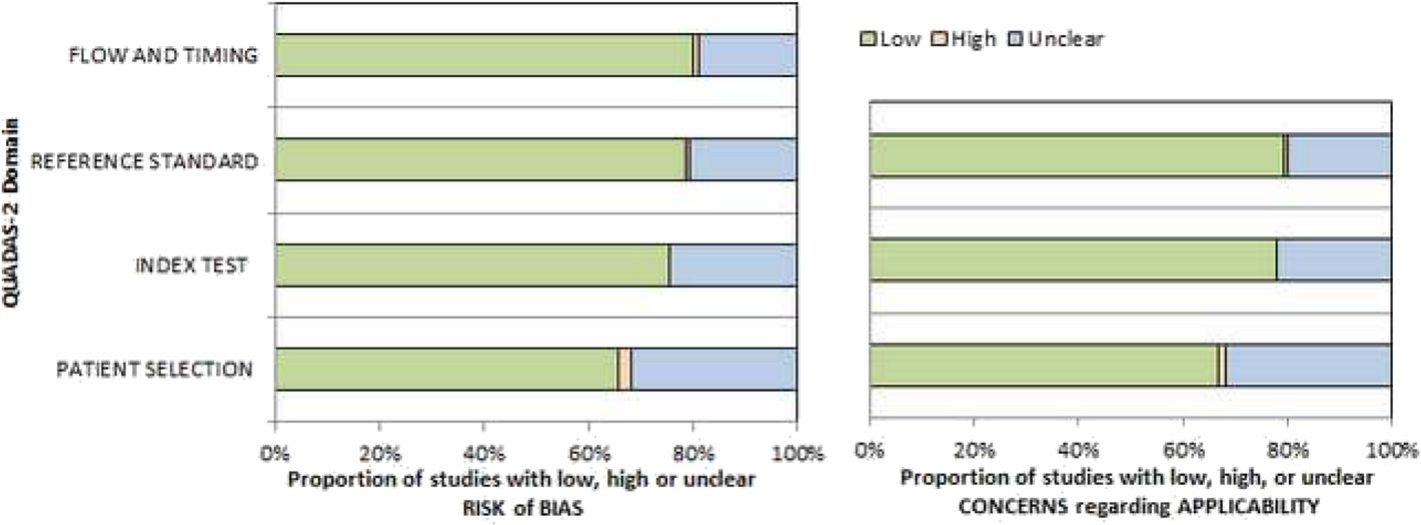
QUADAS-2 quality assessment of the included studies

On the first step, the methodological quality of the 123 studies was checked according to the Quality Assessment of Diagnostic Studies (QUADAS-2) instrument [17] independently by two observers (A.S. and H.J.M.). The results of QUADAS-2 assessment are shown in Fig. 2. The quality of most studies showed an overall low risk of bias.

On the second step, the reported ADC values (mean and standard deviation) were acquired from the papers.

**Fig. 3:**
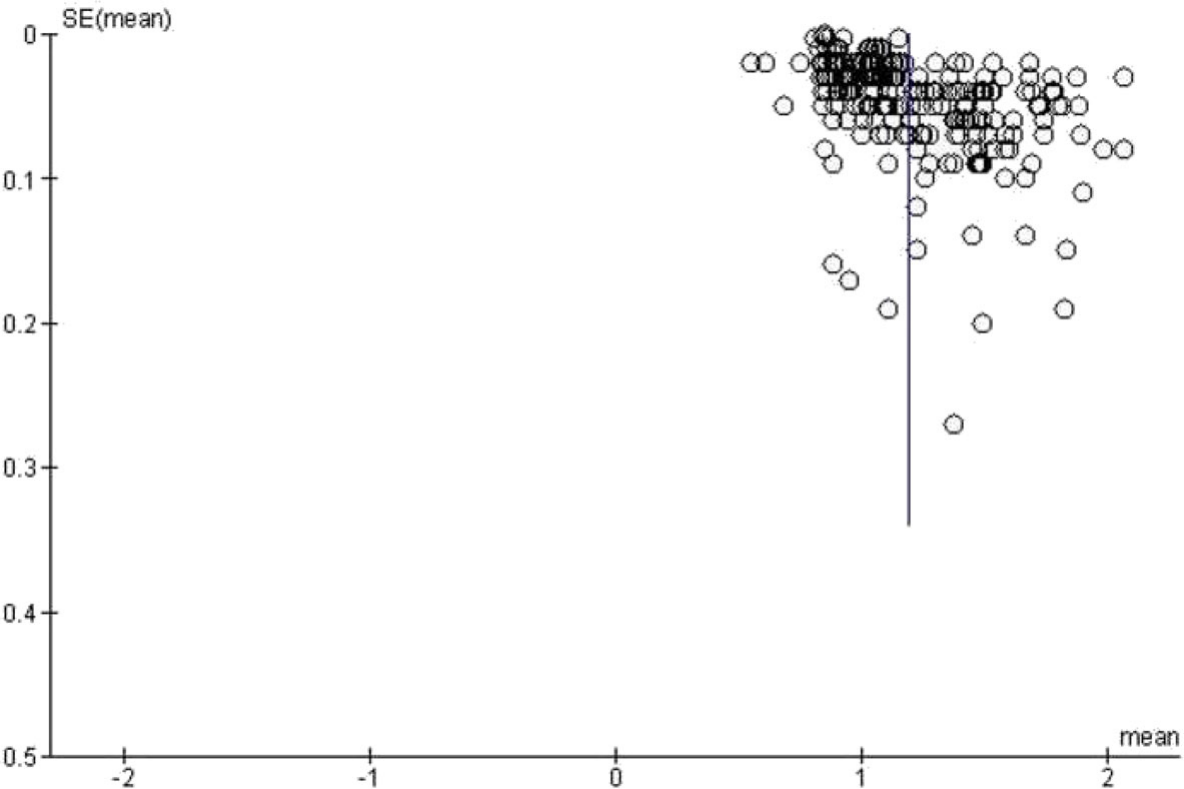
Funnel plot of the publication bias

Thirdly, the meta-analysis was undertaken by using RevMan 5.3 [RevMan 2014. The Cochrane Collaboration Review Manager Version 5.3.]. Heterogeneity was calculated by means of the inconsistency index I^2^ [18, 19]. In a subgroup analysis, studies were stratified by tumor type. In addition, DerSimonian and Laird random-effects models with inverse-variance weights were used without any further correction [20] to account for the heterogeneity between the studies (Fig. 3). Mean ADC values including 95% confidence intervals were calculated separately for benign and malign lesions.

## Results

Of the included 123 studies, 101 (82.1%) were retrospective and 22 (17.9%) prospective (Table 1). The studies represented almost all continents and originated from Asia (*n* = 77, 62.6%), Europe (*n* = 23, 18.7%), North America (*n* = 19, 15.5%), South America (*n* = 3, 2.4%), and Africa (*n* = 1, 0.8%). Different 1.5 T scanners were used in 53 (43.1%) studies, 3 T scanners in 63 reports (51.2%), and in 7 studies (5.7%) both 1.5 and 3 T scanners were used. Overall, 68 studies (55.3%) were performed/reported in the years 2015–2018, 46 studies (37.4%) in the years 2010–2014, and 9 studies (7.3%) in the years 2000–2009.

**Table 1:** Studies inclujded into the meta-analysis

Author, years [Ref.].	Malignant lesions, n	benign lesions, n	Study design	Tesla strength
Akin et al., 2016 [21]	89	92	retrospective	3
An et al., 2017 [22]	112	32	prospective	3
Arponen et al., 2015 [14]	114	23	retrospective	3
Arponen et al., 2018 [23]	25	7	retrospective	3
Baba et al., 2014 [24]	70	13	retrospective	1.5
Baltzer et al., 2010 [25]	54	27	retrospective	1.5
Belli et al., 2015 [26]	289		retrospective	1.5
Belli et al., 2010 [27]	100	26	retrospective	1.5
Bickel et al., 2015 [28]	176		retrospective	3
Bogner et al., 2009 [29]	24	17	retrospective	3
Bokacheva et al., 2014 [30]	26	14	retrospective	3
Çabuk et al., 2015 [31]	22	41	retrospective	1.5
Cai et al., 2014 [32]	149	85	retrospective	1.5
Caivano et al., 2015 [33]	67	43	retrospective	3
Cakir et al., 2013 [15]	30	25	retrospective	3
Chen et al., 2012 [34]	39	18	retrospective	1.5
Chen et al., 2018 [35]	72	44	prospective	3
Cheng et al., 2013 [36]	128	60	retrospective	1.5
Cho et al., 2016 [37]	50	12	retrospective	3
Cho et al., 2015 [38]	38		retrospective	3
Choi et al., 2017 [39]	34		retrospective	3 and 1.5
Choi et al., 2018 [40]	78		prospective	3
Choi et al., 2012 [41]	335		retrospective	1.5
Choi et al., 2017 [42]	221		retrospective	3
Cipolla et al., 2014 [43]	106		retrospective	3
Costantini et al., 2012 [44]	225		retrospective	1.5
Costantini et al., 2010 [45]	162		prospective	1.5
de Almeida et al., 2017 [46]	44	37	retrospective	1.5
Durando et al., 2016 [47]	126		retrospective	3
Eghtedari et al., 2016 [48]	33	18	retrospective	3 and 1.5
Ertas et al., 2016 [49]	85	85	retrospective	3
Ertas et al., 2018 [50]	85	88	retrospective	3
Fan et al., 2018 [51]	126		retrospective	3
Fan et al., 2018 [52]	68	21	retrospective	3
Fan et al., 2017 [53]	82		retrospective	3
Fanariotis et al., 2018 [54]	59	41	retrospective	3
Fornasa et al., 2011 [55]	35	43	retrospective	1.5
Gity et al., 2018 [56]	50	48	prospective	1.5
Guatelli et al., 2017 [57]	161	91	retrospective	1.5
Hering et al., 2016 [58]	25	31	retrospective	1.5
Hirano et al., 2012 [59]	48	27	retrospective	3
Horvat et al., 2018 [60]	218	130	retrospective	3
Hu et al., 2018 [61]	52	36	retrospective	3
Huang et al., 2018 [62]	50	26	prospective	3
Iima et al., 2011 [63]	25		retrospective	1.5
Imamura et al., 2010 [64]	16	11	retrospective	1.5
Inoue et al., 2011 [65]	91	15	retrospective	1.5
Janka et al., 2014 [66]	59	20	retrospective	1.5
Jeh et al., 2011 [67]	155		retrospective	3 and 1.5
Jiang et al., 2018 [68]	171	104	retrospective	1.5
Jiang et al., 2014 [69]	64		retrospective	1.5
Jin et al., 2010 [70]	40	20	retrospective	1.5
Kanao et al., 2018 [71]	79	83	retrospective	3 and 1.5
Kawashima et al., 2017 [72]	137		retrospective	3
Ei Khouli et al., 2010 [73]	101	33	retrospective	3
Kim et al., 2019 [74]	93		retrospective	3
Kim et al., 2018 [75]	121	48	retrospective	3
Kim et al., 2018 [76]	81		retrospective	3
Kim et al., 2009 [77]	60		retrospective	1.5
Kitajima et al., 2018 [78]	67		retrospective	3
Kitajima et al., 2016 [79]	216		retrospective	3
Köremezli Keskin et al., 2018 [80]	59		retrospective	1.5
Kul et al., 2018 [81]	143	70	retrospective	1.5
Kuroki et al., 2004 [82]	55	5	retrospective	1.5
Lee et al., 2016 [83]	128		retrospective	3
Lee et al., 2016 [84]	52		retrospective	3
Li et al., 2015 [85]	55		retrospective	3
Liu et al., 2017 [86]	48	47	retrospective	3
Liu et al., 2015 [87]	176		retrospective	3
Lo et al., 2009 [88]	20	11	prospective	3
Matsubayashi et al., 2010 [89]	26		retrospective	1.5
Min et al., 2015 [90]	29	20	retrospective	1.5
Montemezzi et al., 2018 [91]	453		prospective	3
Mori et al., 2013 [92]	51		retrospective	3
Nakajo et al., 2010 [93]	51		retrospective	1.5
Nogueira et al., 2015 [94]	28	30	prospective	3
Nogueira et al., 2014 [95]	89	68	prospective	3
Ochi et al., 2013 [96]	59	45	retrospective	1.5
Onishi et al., 2014 [97]	17		retrospective	3 and 1.5
Ouyang et al., 2014 [98]	23	16	retrospective	3
Park et al., 2017 [99]	201		retrospective	3
Park et al., 2016 [100]	71		prospective	3
Park et al., 2007 [101]	50		retrospective	1.5
Park et al., 2015 [102]	110		retrospective	3
Parsian et al., 2012 [103]		175	retrospective	1.5
Parsian et al., 2016 [104]		26	retrospective	1.5
Partridge et al., 2018 [105]	242		prospective	3 and 1.5
Partridge et al., 2011 [106]	27	73	retrospective	1.5
Partridge et al., 2010 [107]	29	87	retrospective	1.5
Partridge et al., 2010 [108]	21	91	retrospective	1.5
Pereira et al., 2009 [109]	26	26	prospective	1.5
Petralia et al., 2011 [110]	28		prospective	1.5
Rahbar et al., 2011 [111]	74		retrospective	1.5
Rahbar et al., 2012 [112]	36		retrospective	1.5
Ramírez-Galván et al., 2015 [113]	15	21	prospective	1.5
Razek et al., 2010 [114]	66		prospective	1.5
Roknsharifi et al., 2018 [115]	97	59	retrospective	1.5
Rubesova et al., 2006 [116]	65	25	retrospective	1.5
Sahin et al., 2013 [117]	35	16	retrospective	1.5
Satake et al., 2011 [118]	88	27	retrospective	3
Sharma et al., 2016 [119]	259	67	prospective	1.5
Shen et al., 2018 [120]	71		retrospective	3
Song et al., 2019 [121]	85		retrospective	3
Song et al., 2017 [122]	106	25	prospective	3
Sonmez et al., 2011 [123]	25	20	retrospective	1.5
Spick et al., 2016 [124]	31	24	prospective	3
Spick et al., 2016 [125]	20	84	retrospective	1.5
Suo et al., 2019 [126]	134		retrospective	3
Tang et al., 2018 [127]	54	32	retrospective	3
Teruel et al., 2016 [128]	34	27	prospective	3
Teruel et al., 2016 [129]	38	34	prospective	3
Thakur et al., 2018 [130]	31		retrospective	3
Wan et al., 2016 [131]	74	21	retrospective	1.5
Wang et al., 2016 [132]	31	20	retrospective	3
Woodhams et al., 2009 [133]	204	58	prospective	1.5
Xie et al., 2019 [134]	134		retrospective	3
Yabuuchi et al., 2006 [135]		19	retrospective	1.5
Yoo et al., 2014 [136]	106	63	retrospective	1.5
Youk et al., 2012 [137]	271		retrospective	3 and 1.5
Zhang et al., 2019 [138]	136	74	retrospective	3
Zhao et al., 2018 [139]	25	23	retrospective	3
Zhao et al., 2018 [140]	119	22	retrospective	3
Zhou et al., 2018 [141]	33	39	retrospective	3

**Fig. 4:**
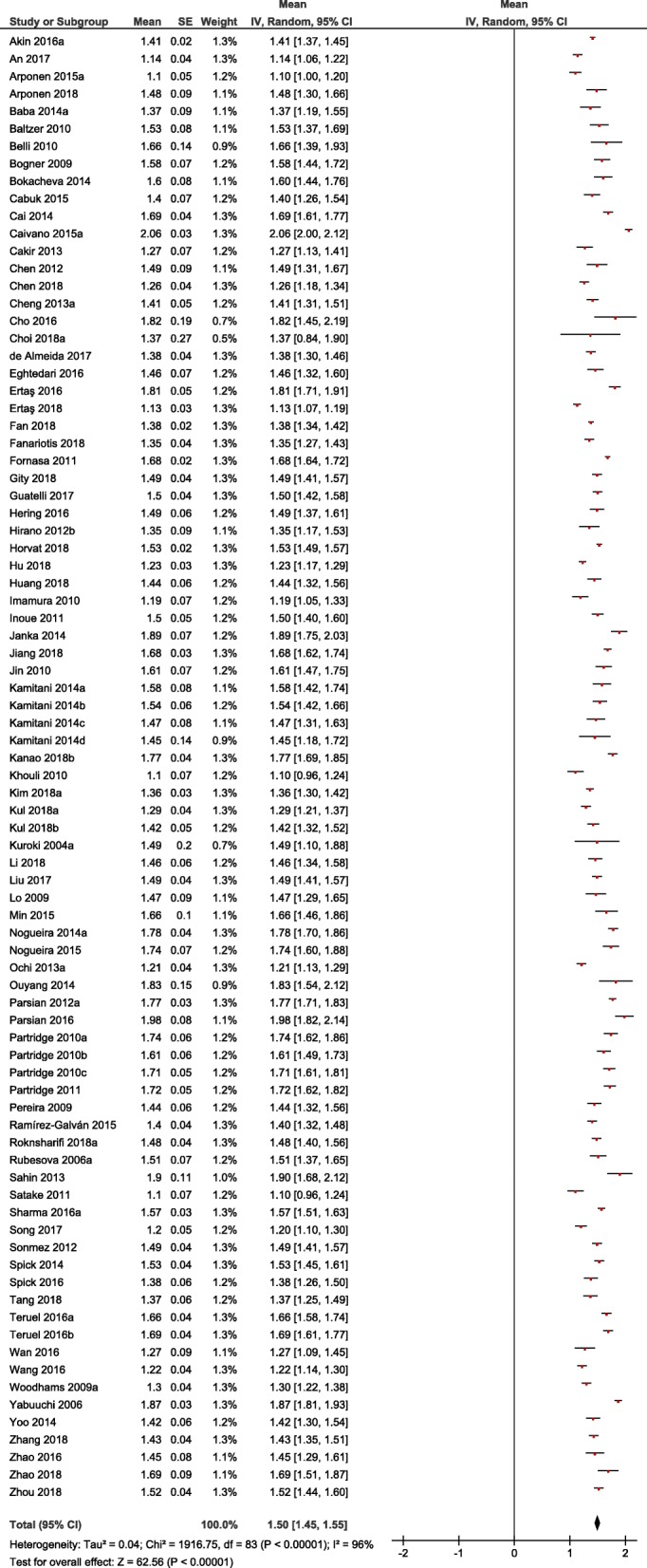
Forrest plots of ADC values reported for benign breast lesions

**Fig. 5:**
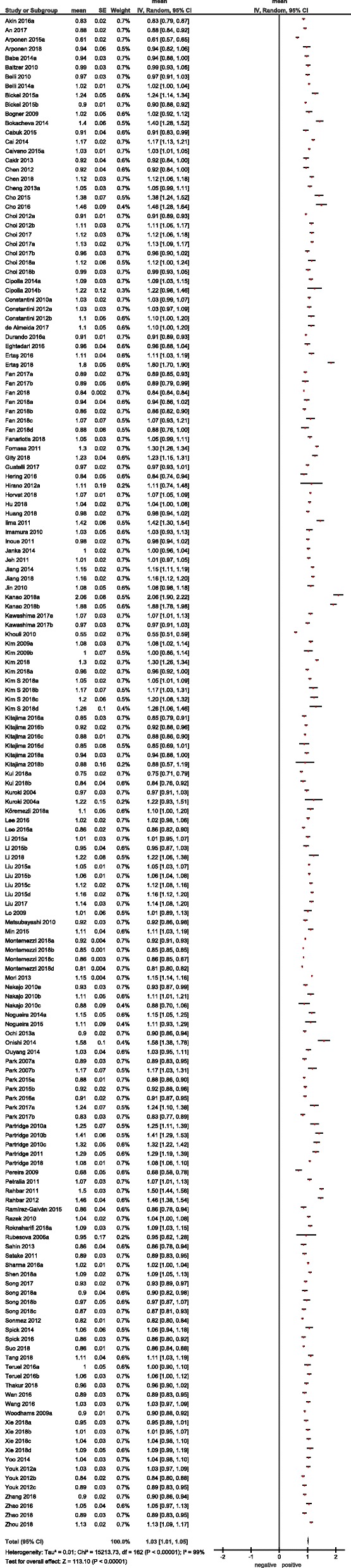
Forrest plots of ADC values reported for malignant breast lesions

**Fig. 6:**
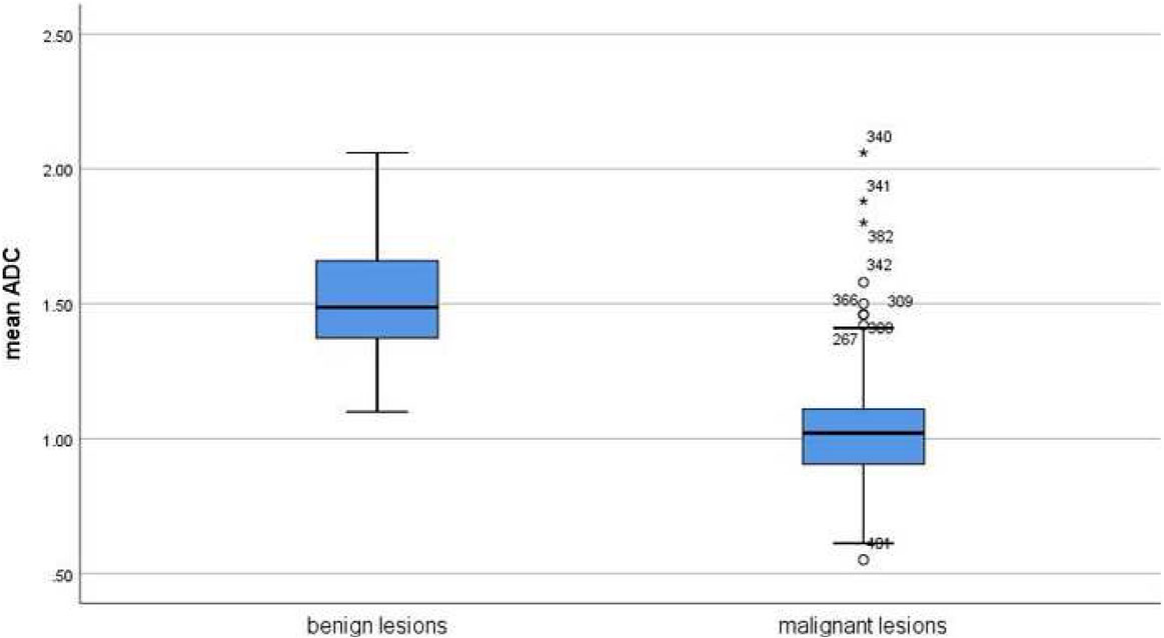
Comparison of ADC values between malignant and benign breast lesions in the overall sample

The acquired 123 studies comprised 13,847 breast lesions. Malignant lesions were diagnosed in 10,622 cases (76.7%) and benign lesions in 3225 cases (23.3%). The mean ADC value of the malignant lesions was 1.03 × 10^− 3^ mm^2^/s and the mean value of the benign lesions was 1.5 × 10^− 3^ mm^2^/s (Figs. 4 and 5). Figure 6 shows the distribution of ADC values in malignant and benign lesions. The ADC values of the two groups overlapped significantly. However, there were no benign lesions under the ADC value of 1.00 × 10^− 3^ mm^2^/s.

**Fig. 7:**
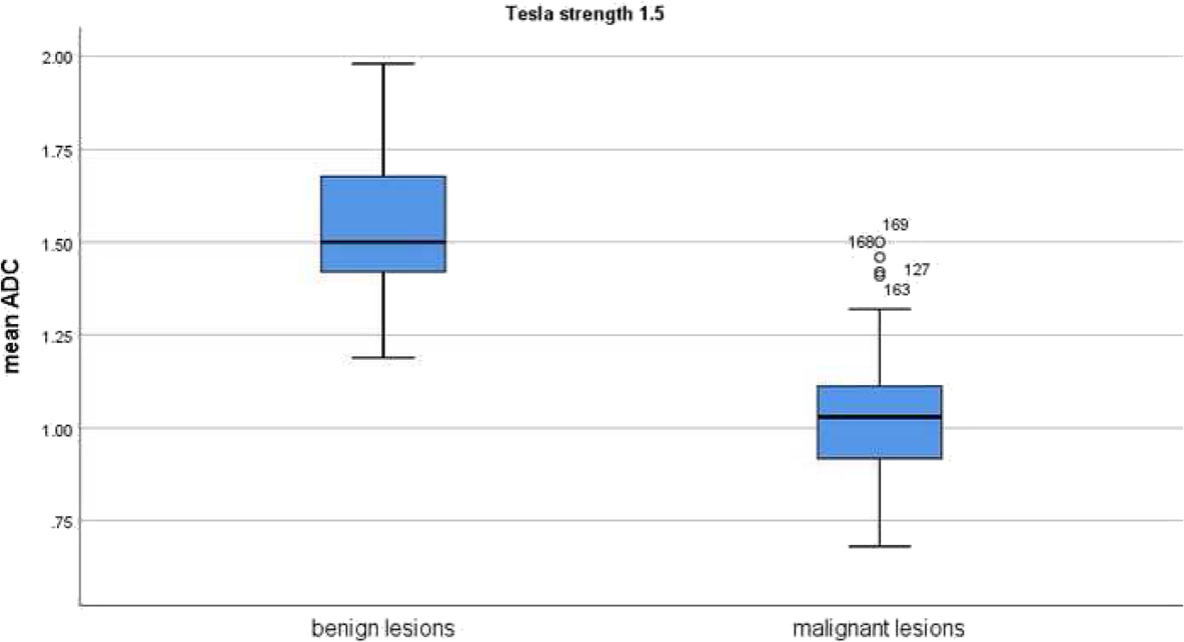
Comparison of ADC values between malignant and benign breast lesions investigated by 1.5 T scanners

On the next step ADC values between malignant and benign breast lesions were compared in dependence on Tesla strength. Overall, 5854 lesions were investigated by 1.5 T scanners and 7061 lesions by 3 T scanners. In 932 lesions, the exact information regarding Tesla strength was not given. In the subgroup investigated by 1.5 T scanners, the mean ADC value of the malignant lesions (*n* = 4093) was 1.05 × 10^− 3^ mm^2^/s and the mean value of the benign lesions (*n* = 1761) was 1.54 × 10^− 3^ mm^2^/s (Fig. 7). The ADC values of the benign lesions were upper the ADC value of 1.00 × 10^− 3^ mm^2^/s.

**Fig. 8:**
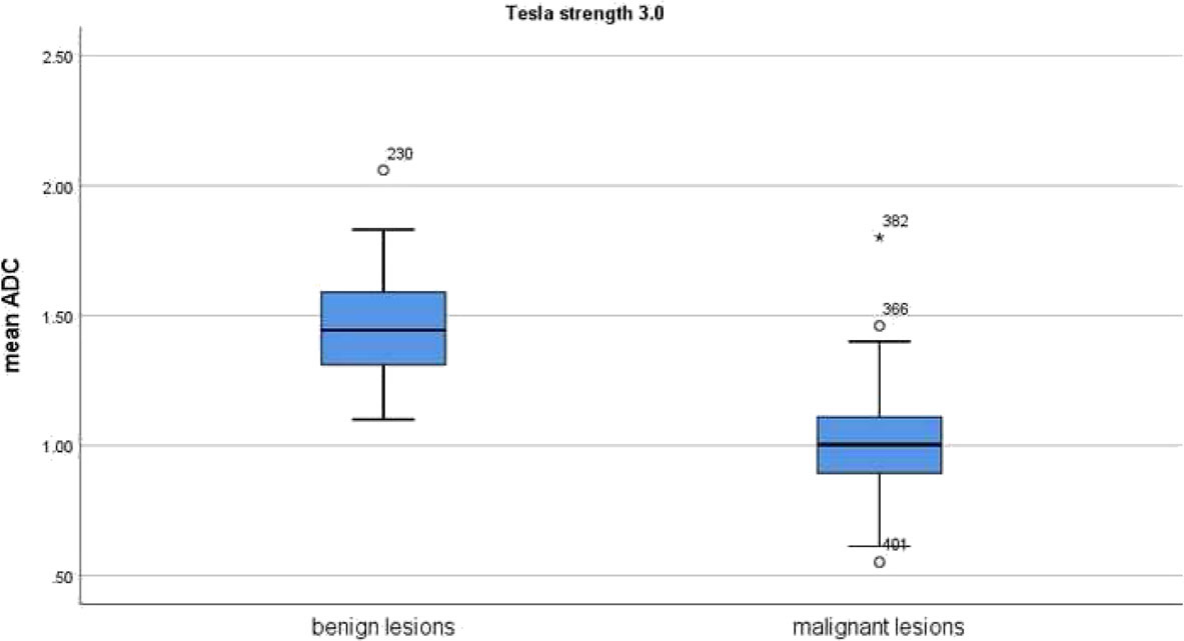
Comparison of ADC values between malignant and benign breast lesions investigated by 3 T scanners

In the subgroup investigated by 3 T scanners, the mean ADC values of the malignant lesions (*n* = 5698) was 1.01 × 10^− 3^ mm^2^/s and the mean value of the benign lesions (*n* = 1363) was 1.46 × 10^− 3^ mm^2^/s (Fig. 8). Again in this subgroup, there were no benign lesions under the ADC value of 1.00 × 10^− 3^ mm^2^/s.

**Fig. 9:**
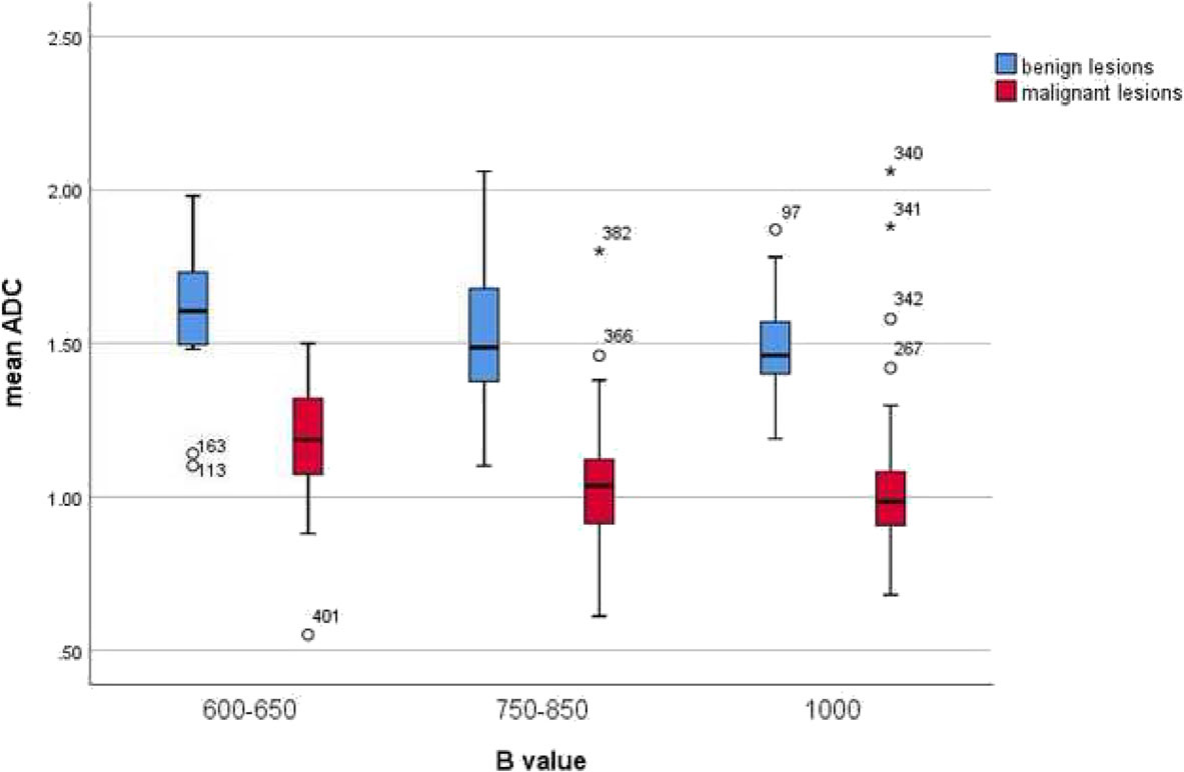
Comparison of ADC values between malignant and benign breast lesions in dependence on the choice of b values

Furthermore, cumulative ADC mean values were calculated in dependence on choice of upper b values. Overall, there were three large subgroups: b600 (426 malignant and 629 benign lesions), b750–850 (4015 malignant and 1230 benign lesions), and b1000 (4396 malignant and 1059 benign lesions). As shown in Fig. 9, the calculated ADC values of benign lesions were over the value 1.00 × 10^− 3^ mm^2^/s in every subgroup.

**Fig. 10:**
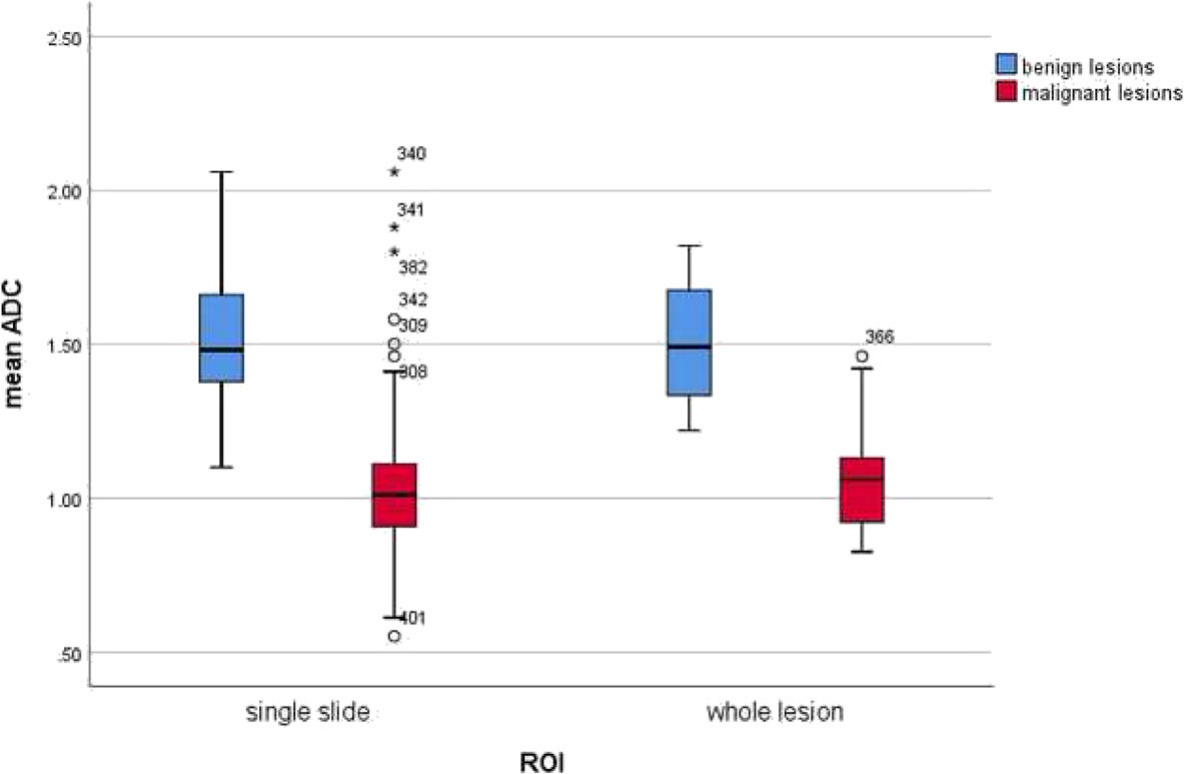
Comparison of ADC values between malignant and benign breast lesions in dependence on measure methods

Finally, ADC values of malignant and benign lesions obtained by single measure in an isolated selected area or ROI (region of interest) and whole lesion measure were analyzed. Single ROI measure was performed for 10,882 lesions (8037 malignant and 2845 benign lesions) and whole lesion analysis was used in 2442 cases (1996 malignant and 446 benign lesions). Also in this subgroup, the ADC values of the benign lesions were above the ADC value of 1.00 × 10^− 3^ mm^2^/s (Fig. 10).

## Discussion

The present analysis investigated ADC values in benign and malignant breast lesions in the largest cohort to date. It addresses a key question as to whether or not imaging parameters, in particular ADC can reflect histopathology of breast lesions. If so, then ADC can be used as a validated imaging biomarker in breast diagnostics. The possibility to stratify breast lesions on imaging is very important and can in particular avoid unnecessary biopsies. As shown in our analysis, previously, numerous studies investigated this question. Interestingly, most studies were reported in the years 2015–2018, which underlines the importance and actuality of the investigated clinical problem. However, as mentioned above, their results were inconsistent. There was no given threshold of an ADC value, which could be used in a clinical setting. Most reports indicated that malignant lesions have lower ADC values than benign findings but there was a broad spectrum of ADC threshold values to discriminate benign and malignant breast lesions. Furthermore, the published results were based on analyses of small numbers of lesions and, therefore, cannot be apply as evident. This limited the possibility to use ADC as an effective diagnostic tool in breast imaging.

Many causes can be responsible for the controversial data. There are no general recommendations regarding use of DWI in breast MRI i.e. Tesla strengths, choice of b values etc. It is known that all the technical parameters can influence DWI and ADC values [142]. Therefore, the reported data cannot apply for every situation. For example, ADC threshold values obtained on 1.5 T scanners cannot be transferred one-to-one to lesions on 3 T.

Furthermore, previous reports had different proportions of benign and malignant lesions comprising various entities. It is well known that some benign breast lesions like abscesses have very low ADC values [143] and some breast cancers, such as mucinous carcinomas, show high ADC values [97, 144]. Furthermore, it has been also shown that invasive ductal and lobular carcinomas had statistically significant lower ADC values in comparison to ductal carcinoma in situ [145]. In addition, also carcinomas with different hormone receptor statuses demonstrate different ADC values [115, 119]. Therefore, the exact proportion of analyzed breast lesions is very important. This suggests also that analyses of ADC values between malignant and benign breast lesions should include all possible lesions. All the facts can explain controversial results of the previous studies but cannot help in a real clinical situation on a patient level basis.

Recently, a meta-analysis about several DWI techniques like diffusion-weighted imaging, diffusion tensor imaging (DTI), and intravoxel incoherent motion (IVIM) in breast imaging was published [146]. It was reported that these techniques were able to discriminate between malignant and benign lesions with a high sensitivity and specificity [146]. However, the authors included only studies with provided sensitivity/specificity data. Furthermore, no threshold values were calculated for discriminating malignant and benign breast lesions. Therefore, no recommendations regarding practical use of DWI in clinical setting could be given.

The present analysis included all published data about DWI findings/ADC values of different breast lesions and, therefore, in contrast to the previous reports, did not have selection bias. It showed that the mean values of benign breast lesions were no lower than 1.00 × 10^− 3^ mm^2^/s. Therefore, this value can be used for distinguishing BC from benign findings. Furthermore, this result is independent from Tesla strength, measure methods and from the choice of b values. This fact is very important and suggests that this cut-off can be used in every clinical situation.

We could not find a further threshold in the upper area of ADC values because malignant and benign lesions overlapped significantly. However, most malignant lesions have ADC values under 2.0 × 10^− 3^ mm^2^/s. As shown, no real thresholds can be found in the area between 1.00 and 2.00 × 10^− 3^ mm^2^/s for discrimination malignant and benign breast lesions.

There are some inherent limitations of the present study to address. Firstly, the meta- analysis is based upon published results in the literature. There might be a certain publication bias because there is a trend to report positive or significant results; whereas studies with insignificant or negative results are often rejected or are not submitted. Secondly, there is the restriction to published papers in English language. Approximately 50 studies could therefore not be included in the present analysis. Thirdly, the study investigated the widely used DWI technique using 2 b-values. However, more advanced MRI sequences, such as intravoxel-incoherent motion and diffusion-kurtosis imaging have been developed, which might show a better accuracy in discriminating benign from malignant tumors. Yet, there are few studies using these sequences and thus no comprehensive analysis can be made.

## Conclusion

An ADC threshold of 1.0 × 10^− 3^ mm^2^/s can be recommended for distinguishing breast cancers from benign lesions. This result is independent on Tesla strength, choice of b values, and measure methods.

## Data Availability

The datasets used and/or analyzed during the current study are available from the corresponding author on reasonable request.

## Abbreviations

ADC: Apparent diffusion coefficient
BC: Breast cancer
MRI: Magnetic resonance imaging
